# Individual and contextual factors associated with orthorexia nervosa tendency during pregnancy

**DOI:** 10.1186/s12884-026-09033-0

**Published:** 2026-04-14

**Authors:** Öznur Bulduk, Özlem Güner

**Affiliations:** 1 Merzifon Karamustafa Paşa Ministiry Hospital, Amasya, Türkiye; 2 Department of Midwifery, Faculty of Health Sciences, Sinop University, Sinop, Türkiye

**Keywords:** Orthorexia nervosa, Pregnancy, Eating behaviors, Antenatal care, Psychosocial factors, Health information seeking

## Abstract

**Background:**

Orthorexia nervosa (ON) refers to a pattern of excessive preoccupation with healthy eating rather than an established clinical diagnosis. Interest in orthorexia-related eating attitudes has increased during pregnancy, a period characterized by heightened nutritional awareness and health-related responsibility. However, evidence regarding the psychosocial, behavioral, and contextual factors associated with such eating attitudes in pregnant women remains limited. This study aimed to examine individual and contextual psychosocial and behavioral factors associated with orthorexia-related eating attitudes during pregnancy.

**Methods:**

This descriptive cross-sectional study was conducted between November 5, 2022 and July 5, 2023 in the obstetrics outpatient clinic and non-stress test unit of a state hospital in Türkiye. A total of 480 pregnant women aged ≥ 18 years with healthy singleton pregnancies participated. Data were collected through face-to-face interviews using the Pregnant Woman Information Form, the Orthorexia Nervosa-11 Scale (ORTO-11), the Pregnancy Self-Perception Scale, and the Brief Psychological Resilience Scale. The ORTO-11 was analyzed as a continuous measure of eating-related attitudes rather than a diagnostic instrument, and lower scores indicate stronger orthorexia-related eating attitudes. Group comparisons were conducted using independent samples t-tests and one-way analysis of variance (ANOVA). Associations between variables were evaluated using Pearson correlation analysis. Variables associated with ORTO-11 scores were examined using hierarchical multiple linear regression analysis.

**Results:**

The mean age of the participants was 28.79 ± 5.02 years, and age was not associated with ORTO-11 scores. Weak correlations were observed between ORTO-11 scores and body mass index and pre-pregnancy weight. Lower ORTO-11 scores (indicating stronger orthorexia-related eating attitudes) were observed among women with higher educational level, persistent nausea and vomiting, discontinuation of dietary supplements, and avoidance of caffeine intake. More frequent pregnancy-related information seeking and discussions with family or friends were also associated with lower ORTO-11 scores. Several psychosocial variables, including pregnancy-related fear, health anxiety, maternal self-perception, partner relationship quality, partner support, and perceived social support, showed weak but statistically significant correlations with ORTO-11 scores. In hierarchical multiple regression analysis, sociodemographic and obstetric variables explained a limited proportion of variance, whereas psychological and behavioral variables contributed additional explanatory value. The final model explained 19.9% of the variance in ORTO-11 scores (adjusted R² = 0.159, *p* < 0.001). After adjustment, higher educational level, higher pregnancy-related fear, lower maternal self-perception, more frequent pregnancy-related information seeking, and changes in caffeine consumption remained independently associated with ORTO-11 scores.

**Conclusion:**

Orthorexia-related eating attitudes during pregnancy appear to occur within a multidimensional biopsychosocial context rather than being explained by obstetric or medical characteristics alone. Given the modest effect sizes and the limited proportion of explained variance, these findings should be interpreted as context-dependent eating-related attitudes rather than evidence of a clinical disorder. Accordingly, the ORTO-11 should not be considered a diagnostic or screening indicator in pregnant populations based on the present data.The findings describe associative patterns observed in a clinical sample, and their clinical relevance and temporal direction require confirmation in longitudinal and population-based studies before any screening or intervention implications can be considered.

## Highlights

Orthorexia-related eating attitudes during pregnancy were evaluated using a multidimensional biopsychosocial framework.

Pregnancy-related fear and frequent pregnancy-related information seeking were associated with lower ORTO-11 scores.

Health-oriented dietary attitudes during pregnancy may be accompanied by more structured or restrictive eating patterns in some women.

The findings indicate that orthorexia-related eating attitudes occur within the psychosocial context of pregnancy.

## Introduction

Pregnancy is a unique period in a woman’s life during which pronounced physiological, psychological, and social changes occur simultaneously. The physical and emotional changes experienced during this period may be accompanied by changes in eating behaviors and may interact with pre-existing vulnerabilities related to eating and weight concerns [1, 2]. Therefore, in the evaluation of the pregnancy process, it is important to consider environmental and cultural factors together with individual characteristics of women [3].

Eating disorders are among the most common psychiatric problems observed in women of reproductive age [4]. During pregnancy, adequate and balanced nutrition, appropriate gestational weight gain, and the maintenance of healthy dietary habits are critically important for both fetal growth and development and maternal health [5]. During this process, pregnant women often tend to improve their dietary patterns and lifestyle behaviors. However, in some women, this tendency may evolve into excessively controlling and restrictive eating behaviors [4]. It has been reported that irregular and unhealthy nutrition may increase the risk of miscarriage, congenital anomalies, and preterm birth in the fetus, while predisposing mothers to health problems such as obesity and diabetes in later life [6]. In addition, pregnant women with a history of eating disorders have been reported to have higher risks of hyperemesis gravidarum, low birth weight, microcephaly, and giving birth to infants small for gestational age [5]. These findings indicate that eating-related patterns during pregnancy are clinically relevant; however, not all pregnancy-related dietary vigilance reflects a diagnosable eating disorder. Some patterns may instead appear as health-focused and behaviorally driven attitudes toward food [7–9].

Beyond clinically defined eating disorders, pregnancy may also be associated with subclinical eating-related patterns characterized by increased attention to health and dietary practices. Orthorexia nervosa has been described as a pattern of excessive preoccupation with healthy eating rather than an established psychiatric disorder [7, 10]. Pregnancy is a period in which engagement with health-related behaviors, including diet, commonly intensifies. For some women, this heightened health orientation may be expressed as more structured or rule-based dietary attitudes. Accordingly, the present study examines orthorexia-related eating attitudes as a continuum of eating-related behaviors during pregnancy. ORTO-11 scores are therefore interpreted as indicators of eating-related attitudes rather than as evidence of clinically significant pathology.

During pregnancy, weight gain and changes in body shape may influence body perception and may be accompanied by increased attention to diet and food choices. Previous studies indicate that concern about healthy eating and body-related perceptions may vary across pregnancy and may be associated with body image experiences [2]. For some women, increased monitoring of dietary intake and bodily changes may coexist with heightened concern regarding food choices and health behaviors [1]. It has been reported that a proportion of women exhibit problematic eating behaviors during pregnancy, and a smaller subgroup meets criteria for clinically defined eating disorders [11].

Nutrition is a central component of antenatal care, and pregnant women commonly receive guidance regarding healthy dietary practices. Within this context, examining how dietary recommendations are perceived and implemented, as well as the psychosocial and behavioral factors accompanying eating-related attitudes, may help to better interpret eating-related behaviors observed in antenatal settings. Therefore, the aim of this study was to examine individual and contextual psychosocial factors associated with orthorexia-related eating attitudes during pregnancy. Because orthorexia nervosa lacks universally accepted diagnostic criteria and validated clinical cut-off values, it was approached as a dimensional behavioral construct rather than a clinical disorder. Accordingly, the ORTO-11 was used as a questionnaire measure of eating-related attitudes rather than a diagnostic instrument, and the findings are interpreted as associative patterns of behavior rather than clinical indicators.

## Methods

### Study design

This study was designed as a descriptive and correlational study to examine individual and contextual psychosocial and informational factors associated with orthorexia-related eating attitudes during pregnancy.

### Study setting and period

The study was conducted between November 5, 2022 and July 5, 2023 in the Obstetrics and Gynecology Outpatient Clinic and the Non-Stress Test (NST) unit of Sinop Atatürk State Hospital, Türkiye, a secondary-care public hospital providing routine antenatal follow-up services for pregnant women in the region.

### Population and sample

The study population consisted of all pregnant women (*N* = 27,283) who attended the Obstetrics and Gynecology Outpatient Clinic and the Non-Stress Test (NST) unit of the hospital for antenatal follow-up between November 2022 and July 2023.

The minimum required sample size was calculated using the finite population proportion formula with a 95% confidence level and a 5% margin of error. Because the prevalence of orthorexia-related eating attitudes in pregnant populations is not well established, the expected prevalence was conservatively set at 50% (*p* = 0.50), which yields the maximum required sample size. Based on this calculation, the minimum sample size was determined to be 379 participants.

Participants were recruited consecutively from women attending the clinic during the data collection period. A total of 480 pregnant women who met the inclusion criteria and agreed to participate were included in the study. The larger sample size was intended to improve estimate precision and to account for potential non-response and incomplete questionnaires.

### Inclusion criteria

Pregnant women were eligible for inclusion if they met the following criteria: age ≥ 18 years; ability to read and write in Turkish; absence of communication barriers; attendance at the outpatient clinic for routine antenatal follow-up; and willingness to participate. Only women with a singleton pregnancy without known obstetric complications were included in order to reduce potential confounding related to high-risk pregnancy conditions.

Women who reported a previously diagnosed psychiatric disorder that could interfere with questionnaire completion were not included. Information regarding psychiatric history was obtained based on participant self-report during the interview.

### Data collection method and procedure

Data were collected by the principal researcher through structured face-to-face interviews conducted in a private area adjacent to the outpatient clinic to ensure confidentiality and minimize external influence. Before the interview, participants were informed about the purpose and procedures of the study, and written informed consent was obtained. Completion of the questionnaires required approximately 20–25 min.

Because of the observational design, blinding to participants’ sociodemographic and obstetric characteristics was not feasible. To minimize interviewer bias, a standardized interview protocol and uniform questioning procedures were followed for all participants.

Information-seeking behavior was defined as the self-reported frequency of obtaining pregnancy-related information (e.g., health, nutrition, or fetal development). Communication with family or friends and partner support variables represented perceived interpersonal interaction during pregnancy and were assessed using structured questionnaire items. These variables were conceptualized as contextual psychosocial factors rather than direct environmental exposures.

### Data collection instruments

The following instruments were used for data collection: (1) Informed Consent Form, (2) Pregnant Woman Information Form, (3) Orthorexia Nervosa-11 Scale (ORTO-11), (4) Pregnancy Self-Perception Scale (PSPS), and (5) Brief Psychological Resilience Scale (BRS).

### Pregnant woman information form

The Pregnant Woman Information Form was developed by the researcher based on the relevant literature and consists of a total of 46 items designed to assess participants’ sociodemographic characteristics, obstetric history, health status during pregnancy, nutrition-related behaviors, and pregnancy-specific characteristics [1, 11].

## Orthorexia Nervosa-11 Scale (ORTO-11)

The ORTO-11 is a scale used to assess obsessive attitudes toward healthy eating. The scale was originally developed by Bratman and Knight [12], and its Turkish validity and reliability study was conducted by Arusoğlu et al. [13]. The ORTO-11 has been used in previous studies involving pregnant populations to examine health-focused eating attitudes and related psychosocial characteristics [2], supporting its contextual application in pregnancy research. The scale consists of 11 items rated on a 4-point Likert scale. Lower total scores indicate stronger orthorexia-related eating attitudes, and all analyses and interpretations were conducted according to this scoring direction. The scale evaluates attitudes related to selecting, purchasing, preparing, and consuming foods perceived as healthy. Internal consistency in this sample was acceptable (Cronbach’s α = 0.71).

### Pregnancy Self-Perception Scale (PSPS)

The Pregnancy Self-Perception Scale was developed by Kumcağız, Ersanlı, and Murat in 2017 [14]. The scale consists of two subdimensions: Perception of Motherhood Related to Pregnancy (7 items) and Perception of Body Related to Pregnancy (5 items), comprising a total of 12 items. Items are scored on a 0–4 point Likert scale. Scores range from 7 to 28 for the motherhood perception subscale and from 5 to 20 for the body perception subscale. The scale is a valid and reliable instrument for assessing pregnant women’s self-perception levels [14].

### Brief Psychological Resilience Scale (BPRS)

The Brief Psychological Resilience Scale was developed by Smith et al. (2008) to assess individuals’ levels of psychological resilience, and its Turkish adaptation was conducted by Doğan (2015) [15, 16]. The scale is a 6-item self-report instrument rated on a 5-point Likert scale. After reversing the negatively worded items, higher total scores indicate higher levels of psychological resilience [16].

### Statistical analysis

Statistical analyses were performed using IBM SPSS Statistics software. Descriptive statistics were presented as mean ± standard deviation for continuous variables and number (n) and percentage (%) for categorical variables. The distribution of continuous variables was evaluated using skewness and kurtosis coefficients; values between − 1.50 and + 1.50 were considered indicative of approximate normality according to Tabachnick and Fidell [17].

For exploratory group comparisons, independent samples t-tests and one-way analysis of variance (ANOVA) were used for normally distributed variables. When significant differences were observed in ANOVA, Tukey post hoc tests were applied. Associations between continuous variables were assessed using Pearson correlation analysis. These univariate analyses were considered descriptive and exploratory, and interpretation of findings was based primarily on the multivariable regression model.

A hierarchical multiple linear regression analysis was conducted to examine factors associated with orthorexia-related eating attitudes during pregnancy. ORTO-11 scores were analyzed as a continuous dependent variable, and no diagnostic cut-off value was applied. Variables were entered into the model in conceptually defined blocks representing sociodemographic/obstetric characteristics, medical history variables, psychological variables, and behavioral/contextual factors. Prior to model interpretation, regression assumptions were evaluated. Multicollinearity was assessed using variance inflation factor (VIF) and tolerance statistics (VIF: 1.02–1.29; tolerance: 0.77–0.99), indicating no multicollinearity. Residual normality was examined using histogram and normal probability plots, and linearity and homoscedasticity were assessed using standardized residual versus predicted value plots; no violations were detected. Influential observations were evaluated using Cook’s distance, and all values were < 1.0. Model fit was evaluated using the coefficient of determination (R²), adjusted R², F-test, and the Durbin–Watson statistic. Statistical significance was set at *p* < 0.05.

### Ethical considerations

Ethical approval was obtained from the Sinop University Human Research Ethics Committee (19 October 2022; approval no. 2022/171). Institutional permission to conduct the study was also obtained from the hospital administration.

All participants were informed about the purpose and procedures of the study, and written informed consent was obtained prior to data collection. Participation was voluntary, and participants were free to withdraw at any time without consequence.

The study was conducted in accordance with the principles of the Declaration of Helsinki. Confidentiality and anonymity were ensured, and data were analyzed in anonymized form.

## Results

Lower ORTO-11 scores indicate stronger orthorexia-related eating attitudes. The mean age of the participants was 28.79 ± 5.02 years, and age was not significantly correlated with ORTO-11 scores (*r* = − 0.052, *p* = 0.260). In contrast, ORTO-11 scores showed weak positive correlations with body mass index (BMI) (*r* = 0.118, *p* = 0.010), pre-pregnancy weight (*r* = 0.105, *p* = 0.022), and duration of marriage (*r* = 0.096, *p* = 0.036) (Table 1).

**Table 1:** Association between sociodemographic and anthropometric characteristics and ORTO-11 scores (*n* = 480)

Variable	Group / Mean ± SD	ORTO-11 Mean ± SD	Test	*p*
Age (years)	28.79 ± 5.02	*r* = − 0.052	Pearson	0.260
BMI (kg/m²)	25.70 ± 5.32	*r* = 0.118	Pearson	0.010*
Pre-pregnancy weight (kg)	67.39 ± 14.81	*r* = 0.105	Pearson	0.022*
Duration of marriage (years)	5.00 ± 4.55	*r* = 0.096	Pearson	0.036*
Education level			ANOVA	< 0.001*
– Primary school (*n* = 22)		29.43 ± 5.55	F = 13.66	
– Secondary school (*n* = 93)		28.88 ± 4.16		
– High school (*n* = 147)		28.21 ± 4.69		
– University/college (*n* = 219)		26.01 ± 4.27		
Partner’s education level			ANOVA	< 0.001*
– Literate (*n* = 11)		28.09 ± 4.46	F = 9.60	
– Primary school (*n* = 37)		28.97 ± 5.56		
– Secondary school (*n* = 107)		28.82 ± 4.63		
– High school (*n* = 143)		27.80 ± 4.27		
– University/college (*n* = 182)		25.87 ± 4.24		
Employment status			t	0.041*
– Not working (*n* = 279)		27.75 ± 4.63	t = 2.05	
– Working (*n* = 201)		26.88 ± 4.54		
Family type			t	0.027*
– Nuclear (*n* = 429)		27.23 ± 4.52	t = − 2.23	
– Extended (*n* = 51)		28.75 ± 5.20		
Smoking status			ANOVA	0.001*
– Yes (*n* = 39)		29.90 ± 3.85	F = 5.40	
– No (*n* = 384)		27.06 ± 4.64		
– Rare / quit (*n* = 57)		27.87 ± 4.34		

Values are presented as mean ± standard deviation unless otherwise indicated. r: Pearson correlation coefficient; t: independent samples t-test; F: one-way analysis of variance (ANOVA). * *p* < 0.05.

A significant difference in ORTO-11 scores was observed according to educational level (F = 13.66, *p* < 0.001). Participants with university/college education had lower mean ORTO-11 scores compared with those with primary, secondary, or high school education. Similarly, partner educational level was associated with ORTO-11 scores (F = 9.60, *p* < 0.001), with lower scores observed when partners had higher education (Table 1).

According to employment status, non-working pregnant women had higher ORTO-11 scores than employed women (t = 2.05, *p* = 0.041). ORTO-11 scores were also higher among women living in extended families compared with those living in nuclear families (t = − 2.23, *p* = 0.027) (Table 1).

Smoking status was associated with ORTO-11 scores (F = 5.40, *p* = 0.001), with the highest mean scores observed among smokers (Table 1).

Gestational week was not significantly correlated with ORTO-11 scores (*r* = 0.049, *p* = 0.282). In contrast, weak positive correlations were observed between ORTO-11 scores and both gravidity (*r* = 0.126, *p* = 0.006) and number of living children (*r* = 0.128, *p* = 0.005) (Table 2).

**Table 2:** Association between pregnancy- and nutrition-related characteristics and ORTO-11 scores(*n* = 480)

Variable	Group / Mean ± SD	ORTO-11 Mean ± SD	Test	*p*
Gestational week	29.67 ± 9.08	*r* = 0.049	Pearson	0.282
Gravidity	1.89 ± 1.07	*r* = 0.126	Pearson	0.006*
Number of living children	0.69 ± 0.83	*r* = 0.128	Pearson	0.005*
Nausea–vomiting			ANOVA	0.030*
– None (*n* = 154)		28.20 ± 4.41	F = 3.54	
– First trimester (*n* = 225)		27.04 ± 4.80		
– Ongoing (*n* = 101)		26.95 ± 4.38		
Spontaneous pregnancy			t	0.001*
– Yes (*n* = 410)		27.69 ± 4.60	t = 3.47	
– No (*n* = 70)		25.64 ± 4.30		
Dietary supplement use			ANOVA	0.009*
– Not using (*n* = 372)		27.74 ± 4.60	F = 3.86	
– Discontinued during pregnancy (*n* = 13)		24.62 ± 3.55		
– Other (*n* = 95)		26.42 ± 4.53		
Caffeine consumption			ANOVA	0.005*
– None (*n* = 65)		25.75 ± 4.36	F = 3.80	
– Rarely (*n* = 189)		27.38 ± 4.61		
– Occasionally / frequently (*n* = 226)		27.89 ± 4.44		
Pregnancy-related information seeking			ANOVA	0.001*
– Never (1)		28.88 ± 5.02	F = 7.59	
– Once a month or less (2)		28.04 ± 4.46		
– 2–3 times per month (3)		28.13 ± 4.51		
– 4 times per month (4)		26.45 ± 4.20		
– ≥4 times per month (5)		25.91 ± 4.32		
Talking with family/friends about pregnancy			ANOVA	0.002*
– Never (1)		28.69 ± 5.43	F = 4.45	
– Once per week or less (2)		28.31 ± 4.47		
– 1–2 times per week (3)		27.61 ± 4.10		
– 3–5 times per week (4)		27.51 ± 4.87		
– ≥5 times per week (5)		26.29 ± 4.59		

Values are presented as mean ± standard deviation unless otherwise indicated. r: Pearson correlation coefficient; t: independent samples t-test; F: one-way analysis of variance (ANOVA). Information-seeking and discussion frequency variables were treated as ordinal categories for group comparisons. * *p* < 0.05.

ORTO-11 scores differed according to the presence of nausea and vomiting during pregnancy (F = 3.54, *p* = 0.030). Participants reporting ongoing nausea and vomiting had lower mean ORTO-11 scores compared with those reporting no symptoms (Table 2).

ORTO-11 scores were higher in spontaneous pregnancies compared with non-spontaneous pregnancies (t = 3.47, *p* = 0.001). Significant group differences were also found according to dietary supplement use (F = 3.86, *p* = 0.009), with the lowest mean scores observed among women who discontinued supplement use during pregnancy (Table 2).

Caffeine consumption frequency was associated with ORTO-11 scores (F = 3.80, *p* = 0.005). The lowest mean scores were observed among participants who reported no caffeine consumption (Table 2).

The frequency of pregnancy-related information seeking was associated with ORTO-11 scores (F = 7.59, *p* = 0.001). Participants who reported never seeking information had higher mean scores than those reporting more frequent information seeking. Similarly, ORTO-11 scores differed according to the frequency of discussing pregnancy-related issues with family or friends (F = 4.45, *p* = 0.002), with lower mean scores observed among those reporting more frequent discussions (Table 2).

Lower ORTO-11 scores indicate stronger orthorexia-related eating attitudes.

A weak but statistically significant negative correlation was observed between maternal perception and ORTO-11 scores (*r* = − 0.099, *p* = 0.031). No statistically significant correlations were found between body perception or psychological resilience and ORTO-11 scores (*p* > 0.05) (Table 3).

**Table 3:** Associations between psychosocial variables, perception scales, and ORTO-11 scores

Variable	Mean ± SD	Correlation with ORTO-11 (*r*)	*p*
Maternal perception	26.01 ± 3.02	−0.099	0.031*
Body perception	9.50 ± 3.79	−0.054	0.237
Psychological resilience	18.76 ± 2.73	0.065	0.155
Fear related to pregnancy	5.51 ± 2.92	−0.167	< 0.001*
Health anxiety	4.07 ± 2.48	−0.124	0.006*
Partner relationship quality	9.01 ± 1.70	−0.122	0.007*
Partner support	8.89 ± 2.00	−0.138	0.002*
Social support	7.42 ± 2.40	−0.116	0.011*

Values are presented as mean ± standard deviation. r: Pearson correlation coefficient. Lower ORTO-11 scores indicate stronger orthorexia-related eating attitudes. * *p* < 0.05.

Pregnancy-related fear and health anxiety were negatively correlated with ORTO-11 scores (*r* = − 0.167, *p* < 0.001 and *r* = − 0.124, *p* = 0.006, respectively). A negative correlation was also observed between partner relationship quality and ORTO-11 scores (*r* = − 0.122, *p* = 0.007).

In addition, weak negative correlations were found between ORTO-11 scores and partner support (*r* = − 0.138, *p* = 0.002) and perceived social support (*r* = − 0.116, *p* = 0.011) (Table 3).

A hierarchical multiple linear regression analysis was performed to examine variables related to ORTO-11 scores during pregnancy (Table 4).

In the first block, sociodemographic and obstetric variables (age, education level, gestational week, number of pregnancies, and body mass index) accounted for 9.3% of the variance in ORTO-11 scores (R² = 0.093, *p* < 0.001). The addition of medical variables (psychiatric history and chronic disease) produced a small increase in explained variance (R² = 0.102; ΔR² = 0.009).

After inclusion of psychological variables (pregnancy-related fear, pregnancy-related anxiety, psychological resilience, and pregnancy self-perception), the explained variance increased to 14.8% (ΔR² = 0.046, *p* < 0.001). The final block, including behavioral and contextual variables (pregnancy-related information-seeking behavior, caffeine consumption change, and supplement use), increased the explained variance to 18.1% (Adjusted R² = 0.149, F(18,461) = 5.656, *p* < 0.001). The Durbin–Watson statistic was 2.08 (Tablo 4).

In the final model, age (B = − 0.093, *p* = 0.048), education level (primary, secondary, and high school vs. university), pregnancy-related fear (B = − 0.188, *p* = 0.012), pregnancy self-perception (B = − 0.093, *p* = 0.041), pregnancy-related information-seeking frequency (B = − 0.437, *p* = 0.005), and caffeine consumption change (decreased or increased intake) were statistically significant predictors of ORTO-11 scores. Gestational week, number of pregnancies, body mass index, chronic disease, psychiatric history, and supplement use were not statistically significant in the adjusted model (Tablo 4).

**Table 4:** Hierarchical multiple regression analysis of factors associated with ORTO-11 scores during pregnancy

Variable	B	SE	β	t	*p*	95% CI
Age	−0.093	0.047	−0.101	−1.986	0.048	−0.184 – −0.001
Primary education	2.829	1.081	0.126	2.616	0.009	0.704–4.953
Secondary education	2.302	0.608	0.197	3.784	< 0.001	1.107–3.497
High school education	1.582	0.503	0.158	3.143	0.002	0.593–2.571
Gestational week	0.014	0.022	0.027	0.622	0.534	−0.030–0.058
Number of pregnancies	0.105	0.242	0.024	0.433	0.665	−0.370–0.580
Body Mass Index (BMI)	0.067	0.038	0.077	1.741	0.082	−0.009–0.143
Model 1: *R* = 0.305; R² = 0.093; Adjusted R² = 0.079; F = 6.899; *p* < 0.001						
Chronic disease	1.118	0.635	0.077	1.759	0.079	−0.131–2.367
Psychiatric history	−0.660	1.034	−0.028	−0.638	0.524	−2.691–1.372
Model 2: *R* = 0.319; R² = 0.102; ΔR²= 0.009; Adjusted R² = 0.085; F = 5.912; *p* < 0.001						
Fear of pregnancy	−0.188	0.074	−0.119	−2.526	0.012	−0.334 – −0.042
Pregnancy-related anxiety	−0.165	0.094	−0.081	−1.754	0.080	−0.350–0.020
Psychological resilience	0.091	0.074	0.054	1.226	0.221	−0.055–0.237
Self-perception	−0.093	0.045	−0.092	−2.054	0.041	−0.182 – −0.004
Model 3: *R* = 0.385; R² = 0.148; ΔR²= 0.046; Adjusted R² = 0.124; F = 6.229; *p* < 0.001						
Seeking pregnancy-related information	−0.437	0.154	−0.137	−2.839	0.005	−0.739 – −0.134
Decreased caffeine intake	1.472	0.590	0.138	2.494	0.013	0.312–2.632
Increased caffeine intake	1.928	0.813	0.133	2.372	0.018	0.331–3.526
Discontinued supplement use	0.861	0.831	0.074	1.036	0.301	−0.772–2.494
Initiated supplement use	0.355	0.957	0.026	0.371	0.711	−1.526–2.236
Model 4: *R* = 0.425; R² = 0.181; ΔR²= 0.033; Adjusted R² = 0.149; F = 6.229; *p* < 0.001; Durbin-Watson = 2.08						

Dependent variable: ORTO-11 total score (continuous). Lower scores indicate stronger orthorexia-related eating attitudes. Categorical variables were entered using dummy coding. For education level, university education was the reference category. Psychiatric disease and chronic disease were coded as 0 = no and 1 = yes. For caffeine consumption change, the reference category was “no change in caffeine intake. For supplement use, the reference category was participants who reported regular supplement use during pregnancy

Continuous variables (age, gestational week, number of pregnancies, BMI, pregnancy-related fear, pregnancy-related anxiety, psychological resilience, pregnancy self-perception, and pregnancy-related information-seeking frequency) were entered as scale variables

Lower ORTO-11 scores represent stronger orthorexia-related eating attitudes; therefore, negative regression coefficients indicate lower ORTO-11 scores.

## Discussion

The present study examined orthorexia-related eating attitudes during pregnancy within a multidimensional framework including sociodemographic, obstetric, psychological, and behavioral variables. The hierarchical regression model showed that obstetric and medical characteristics explained a limited proportion of the variability in ORTO-11 scores, whereas additional variance was observed after the inclusion of psychological and contextual behavioral variables. These findings indicate that orthorexia-related eating attitudes during pregnancy were not limited to pregnancy physiology or medical status but were observed together with health perceptions, monitoring behaviors, and engagement with pregnancy-related information. Given the cross-sectional design, these findings should be interpreted as associative relationships reflecting concurrent behavioral patterns rather than directional or causal effects.

Regarding anthropometric characteristics, weak but statistically significant correlations were observed between ORTO-11 scores and body mass index (BMI) and pre-pregnancy weight. Because lower ORTO-11 scores indicate stronger orthorexia-related eating attitudes, lower BMI values were associated with lower ORTO-11 scores within the sample. However, the magnitude of these correlations was small, suggesting limited practical relevance. Previous studies have reported inconsistent findings regarding the relationship between BMI and orthorexia-related eating attitudes [18, 19], and heterogeneous associations with body image and weight-related concerns have also been described [20, 21]. During pregnancy, increased attention to diet and weight monitoring has been reported in the literature, and such characteristics may occur together with health-focused eating attitudes. Nevertheless, given the cross-sectional design, these findings should be interpreted as co-occurring characteristics rather than evidence of a directional relationship.

Pregnancy-related experiences and physical conditions were also associated with ORTO-11 scores. ORTO-11 scores were higher with increasing parity, indicating weaker orthorexia-related eating attitudes among women with a greater number of previous pregnancies. Previous studies have reported limited and inconsistent findings regarding the relationship between parity and orthorexia-related eating attitudes [4, 19]. Participants reporting persistent nausea and vomiting during pregnancy also had lower ORTO-11 scores. Rather than suggesting a directional effect, this finding indicates that pregnancy-related physical symptoms and eating-related attitudes were observed concurrently in this sample. Reports of higher rates of hyperemesis gravidarum among women with eating disorder histories [22] further support that such characteristics may co-occur during pregnancy. Similarly, lower ORTO-11 scores observed among women with non-spontaneous pregnancies should be interpreted cautiously, as the cross-sectional design does not allow determination of temporal ordering. Overall, these findings indicate concurrent associations between eating-related attitudes and pregnancy experiences, and the cross-sectional design does not permit inference regarding direction or causality.

Regarding psychological factors, weak but statistically significant correlations were observed between pregnancy-related fear, health anxiety, and ORTO-11 scores. Higher fear and anxiety levels were accompanied by lower ORTO-11 scores, indicating stronger orthorexia-related eating attitudes in bivariate analyses. However, in the multivariable model, pregnancy-related fear remained associated with ORTO-11 scores, whereas other psychological variables did not retain statistical significance after adjustment. These findings indicate that orthorexia-related eating attitudes and pregnancy-related concerns co-occur within the same period rather than representing a specific psychological pathway. Similar co-occurrence of health concerns and health-focused eating attitudes has been described in previous literature [7]. Nevertheless, these findings should be interpreted as concurrent associations, and the cross-sectional design does not allow inference regarding temporal or directional relationships.

Maternal perception scores also showed a weak association with ORTO-11 scores in bivariate analyses, indicating that maternal self-perception and eating-related attitudes may occur together during pregnancy. Previous qualitative studies describe pregnancy as a period characterized by increased attention to health-promoting behaviors [23, 24], and some research has noted greater structuring of health-related practices during this time [25]. Expectations related to the “good mother” role have also been documented in the literature [26–28]. However, maternal perception did not retain an independent association after adjustment for other psychological and behavioral variables in the multivariable model. Accordingly, maternal perception may reflect a component of the broader psychosocial context surrounding pregnancy rather than a separate associated variable. No temporal or directional interpretation can be made.

Information seeking and social interaction were also associated with ORTO-11 scores. More frequent pregnancy-related information seeking and discussions with family or friends were accompanied by lower ORTO-11 scores, corresponding to stronger orthorexia-related eating attitudes in the sample. Previous literature has described increased attention to nutrition-related information and communication during pregnancy [29, 30]. Differences in ORTO-11 scores were also observed according to partner relationship quality and perceived social support. These patterns may reflect the social context in which dietary decisions are discussed and negotiated during pregnancy. In this context, partner involvement may be accompanied by reassurance, shared responsibility for fetal health, and, in some cases, encouragement of cautious or rule-based dietary practices. Such observations should be interpreted as contextual characteristics rather than behavioral mechanisms, and similar associations between interpersonal context and health-focused eating attitudes have been reported in previous research [8, 9, 31, 32]. Overall, information seeking and interpersonal communication during pregnancy appear to represent concurrent behavioral patterns within the psychosocial environment of pregnancy rather than discrete risk or protective factors. Consistent with the cross-sectional design, no temporal or causal inference can be made.

Certain nutrition-related health behaviors were also associated with ORTO-11 scores. Lower ORTO-11 scores were observed among pregnant women who reported discontinuing dietary supplements, avoiding caffeine consumption, and not smoking. Evidence regarding supplement use and caffeine consumption in relation to orthorexia-related eating attitudes during pregnancy is limited; however, previous studies have reported differences in dietary restriction patterns among individuals with health-focused eating attitudes or eating disorder histories [4, 33].

Importantly, these findings should be interpreted within the prenatal context. During pregnancy, avoidance of specific foods or substances often represents precautionary health behavior and adherence to medical advice rather than maladaptive restriction. Therefore, lower ORTO-11 scores in this context may reflect increased health vigilance and dietary monitoring rather than clinically impairing behavior. Accordingly, these variables are better interpreted as contextual behavioral correlates of eating-related attitudes measured by the ORTO-11 rather than indicators of a defined eating disorder pattern. Because of the cross-sectional design, the temporal or explanatory direction of these relationships cannot be determined.

Within the sociodemographic context, educational level was associated with ORTO-11 scores. Lower ORTO-11 scores were observed among pregnant women with university/college education and among those whose partners had higher educational levels. Because lower ORTO-11 scores indicate stronger orthorexia-related eating attitudes, this pattern may reflect increased attention to diet and greater engagement with health recommendations among more educated women rather than a clinically meaningful eating pathology.

Previous studies have reported heterogeneous findings regarding the relationship between education and orthorexia-related eating attitudes [18, 19, 34], and similar variability has been described across different populations [35, 36]. Higher educational attainment is often associated with greater health literacy and more active information seeking, which may increase dietary monitoring during pregnancy. Differences in ORTO-11 scores were also observed according to family structure; however, these findings should be interpreted as sociodemographic correlates rather than behavioral or psychological determinants. Given the weak magnitude of the associations and the cross-sectional design, these variables are better considered contextual descriptive characteristics rather than explanatory factors.

The hierarchical modeling approach provided additional context for interpreting the findings. When sociodemographic and obstetric variables were entered alone, they explained only a limited proportion of the variance in ORTO-11 scores, whereas the inclusion of psychological and behavioral variables increased the explained variance. This pattern suggests that orthorexia-related eating attitudes during pregnancy are less closely related to pregnancy physiology or medical status and more closely associated with health-related perceptions and behavioral engagement. Previous literature has described orthorexia nervosa not as a conventional eating disorder but as a pattern characterized by excessive health orientation, dietary vigilance, and cognitive preoccupation with food purity [7, 10]. Studies have also reported that orthorexia-related attitudes are associated with health anxiety, perceived responsibility for health, and information-seeking behaviors rather than with metabolic or anthropometric characteristics. The present findings are consistent with these conceptualizations, as psychological and behavioral variables explained more variability than obstetric or medical characteristics.

Pregnancy represents a period of intensified health monitoring and exposure to normative dietary recommendations. Antenatal care commonly emphasizes nutritional responsibility for fetal well-being, which may increase attention to diet and perceived obligation to follow precautionary practices. Previous research has shown that pregnant women frequently modify dietary behaviors in response to perceived fetal vulnerability and health messaging, sometimes adopting restrictive or rule-based eating patterns. In this context, orthorexia-related eating attitudes may reflect heightened health vigilance rather than pathology.

The association with information-seeking behavior and interpersonal communication further supports this interpretation. Increased engagement with health information during pregnancy has been linked to both adaptive coping and heightened anxiety regarding fetal health. Therefore, frequent monitoring, communication, and precautionary dietary practices may emerge as behavioral responses to uncertainty rather than indicators of a distinct eating disorder.

In addition, nutrition during pregnancy is strongly emphasized in healthcare communication, and socially desirable responding may influence how women report health-related eating behaviors. Consequently, ORTO-11 scores may partly capture normative health vigilance and perceived expectations within antenatal care rather than solely individual behavioral tendencies.

Although the overall regression model was statistically significant and explained approximately 18% of the variance in ORTO-11 scores, the magnitude of the observed associations was small. Behavioral constructs in psychosocial research often demonstrate modest explained variance, and statistical significance should not be interpreted as clinical relevance. Accordingly, the identified variables should be considered correlates of eating-related attitudes rather than predictive determinants.

Because of the cross-sectional design, the temporal order of these relationships cannot be determined. The associations represent concurrent characteristics measured at a single time point, and bidirectional or context-dependent relationships are possible. The findings therefore do not support causal inference and should be interpreted as descriptive of behavioral patterns observed within a clinical antenatal population.

### Strengths and limitations

One of the major strengths of this study is its relatively large sample size and the multidimensional evaluation of orthorexia-related eating attitudes during pregnancy. By examining sociodemographic, obstetric, and psychosocial variables simultaneously, the study situates orthorexia-related eating attitudes within a broader contextual framework rather than limiting interpretation to nutritional behaviors alone. The use of hierarchical multiple linear regression further allowed assessment of variables independently associated with ORTO-11 scores, providing a more comprehensive interpretation of the observed associations. In addition, studies addressing pregnancy-specific psychosocial characteristics together with orthorexia-related eating attitudes are limited, and this study contributes to this emerging area of research.

Several limitations should also be considered. First, the cross-sectional design precludes causal inference and does not allow determination of temporal direction between variables. Second, participants were recruited from a single state hospital using a convenience sampling approach; therefore, the sample primarily represents pregnant women attending outpatient and NST services in this specific clinical setting. Healthcare utilization patterns and local sociocultural characteristics may have influenced participation, and the findings should be interpreted cautiously with respect to generalizability beyond similar clinical populations.

All data were collected using self-report questionnaires administered in a face-to-face clinical context. Consequently, recall bias, shared-method variance, and social desirability bias may have affected responses. During pregnancy, adherence to recommended health behaviors—particularly nutritional practices—is strongly emphasized in antenatal care. Participants may therefore have over-reported cautious or health-oriented eating behaviors in line with perceived expectations of healthcare providers. Accordingly, some lower ORTO-11 scores may reflect conformity to normative maternal health expectations rather than solely individual eating-related tendencies.

Finally, although the ORTO-11 demonstrated acceptable internal consistency in this sample (Cronbach’s α = 0.71), the instrument has recognized conceptual and psychometric limitations. No universally accepted diagnostic framework exists for orthorexia nervosa, and distinguishing health-oriented vigilance from maladaptive rigidity remains challenging. Therefore, the ORTO-11 should not be interpreted as a diagnostic tool, and the findings are best understood as reflecting eating-related attitudes and behavioral tendencies rather than clinically significant pathology.

### Clinical and public health implications

The findings of this study suggest that increased attention to healthy eating during pregnancy may, in some individuals, be accompanied by more rule-oriented dietary attitudes. These observations should be interpreted within the psychosocial context of pregnancy, a period in which responsibility toward fetal health, dietary caution, and active information seeking are strongly encouraged.

Because of the cross-sectional design, the study does not allow causal inference, establishment of clinically actionable cut-off values, or prediction of maternal or fetal outcomes. Accordingly, ORTO-11 scores should not be used for clinical screening, diagnostic classification, or intervention planning. Rather, the results indicate that health-focused eating attitudes during pregnancy may represent context-dependent behavioral patterns that can overlap with pregnancy-related concern and anxiety.

Lower ORTO-11 scores in pregnant populations therefore require cautious interpretation, as they may reflect normative health responsibility rather than pathological behavior. Such attitudes may be clinically relevant primarily when accompanied by psychological distress, functional impairment, or nutritional inadequacy.

Longitudinal and outcome-based studies are needed to determine whether these attitudes have predictive value for maternal well-being or perinatal outcomes and to clarify their distinction from general anxiety and pregnancy-related fear. No clinical recommendations are made based on the present findings.

## Conclusion

Orthorexia-related eating attitudes during pregnancy were associated with several sociodemographic, psychological, and behavioral characteristics. The hierarchical modeling indicated that these attitudes were more closely linked to psychosocial and health-behavior–related factors than to obstetric or medical characteristics. Given the small effect sizes and limited explained variance, the findings should be interpreted as reflecting context-dependent eating-related attitudes rather than a distinct clinical condition. Accordingly, the ORTO-11 should not be considered a diagnostic indicator in pregnant populations but a measure of eating-related attitudes within the antenatal context. Because of the cross-sectional design, causal or temporal relationships cannot be determined. Further longitudinal and qualitative studies are required to clarify the development, persistence, and potential clinical relevance of these attitudes. The findings may contribute to a better understanding of eating-related attitudes within similar clinical settings; however, confirmation in multicenter and population-based studies is necessary before broader generalization.

## Data Availability

The datasets used and/or analysed during the current study are available from the corresponding author on reasonable request.
